# Potential Inflammatory Biomarker in Patients with Attention Deficit Hyperactivity Disorder

**DOI:** 10.3390/ijms232113054

**Published:** 2022-10-27

**Authors:** Ji Hyun Park

**Affiliations:** College of Pharmacy, Duksung Women’s University, Seoul 01369, Korea; pharmerpark@duksung.ac.kr

**Keywords:** kynurenine pathway, inflammatory biomarkers, inflammation, ADHD, neurobiology, kynurenine

## Abstract

Attention deficit hyperactivity disorder (ADHD) is a prevalent neurodevelopmental disorder that can diminish the quality of life of both children and adults in academic, occupational, and social contexts. The kynurenine pathway (KP) contains a set of enzymatic reactions involved in tryptophan (TRP) degradation. It is known to be associated with the risk of developing ADHD. This review will address the KP and underlying mechanism of inflammation in ADHD. Potential inflammatory biomarkers reported in the most recent studies are summarized. Although a strong neuroimmunological basis has been established due to the advances of recent neurobiological research, the pathophysiology of ADHD remains unclear.

## 1. Introduction

Attention deficit hyperactivity disorder (ADHD), one of the most commonly found neurodevelopmental disorders in children and adolescents, is characterized by impairing symptoms of inattention, hyperactivity, and impulsivity [1]. ADHD affects 3–10% of school-aged children, with a reported prevalence rate of 5.9 to 8.6% in the Korean population [2]. Its prevalence varies among studies. In a study in Taiwan, the local prevalence was estimated to be 7.5% [3]. In a Norwegian study of children aged 8 to 10 years, its prevalence was 1.7% [4]. A meta-analysis reported that the worldwide prevalence of ADHD in children and adolescents was 5.3% (95% CI: 5.01–5.56) in 2007 [5]. It has been shown that 60–85% of ADHD cases diagnosed in children persist into adulthood [6]. Consistently, a meta-analysis has reported that the prevalence of ADHD in adults is 2.4% [7].

ADHD has been associated with distinctively worse school performance independent from socioeconomic factors among children [8]. It is also associated with significant impairment of occupational, academic, and social functioning in adults [9]. According to the DSM-5 criteria, the diagnosis of ADHD in children is based on the presence of at least six out of nine symptoms in two areas of inattention and hyperactivity impulsivity with observed behavioral issues [10,11]. The current diagnostic criteria of ADHD rely on subjective reporting from patients or other informants (patients, teachers) and clinical observations, which might not be able to differentiate definite illness from normal variation [12]. Meanwhile, public awareness and widespread recognition of this disorder have led to an obvious increase in the diagnosis and treatment rate of adult ADHD over the last decade [13]. Overdiagnosis and misdiagnosis are also a concern as they might result in unnecessary labeling, extra costs for excessive tests, unneeded therapies, and increased healthcare costs [14].

**Figure 1:**
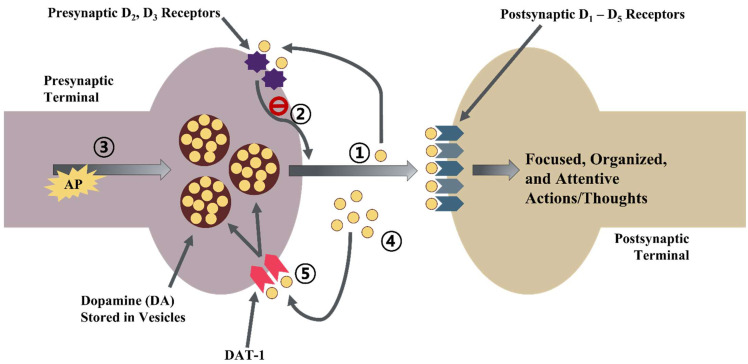
**Integration of catecholamine hypothesis of ADHD.** ① Dopamine (yellow circles) acts on postsynaptic D1-D5 receptors. D2 and D3 receptors are locally positioned on the presynaptic neuron. Small amounts of dopamine stored in vesicles are released into the synapse from the presynaptic terminal if there is no action potential. ② This composes a tonic pool that acts on D2 and D3 presynaptic receptors, providing feedback to inhibit the release of DA. ③ Action potential arrives at the presynaptic terminal. ④ A large amount of vesicular DA is released into the synapse. This composes the phasic pool acting on postsynaptic receptors. The amount of DA released in the phasic pool depends on feedback inhibition, which is fired by the stimulation of D2 and D3 receptors of the tonic pool. ⑤ DAT-1 on the presynaptic terminal reuptakes DA and the action of DA on the postsynaptic receptor is terminated. Similar action of NE and NE receptors (α and β) is shown in the PFC. It is postulated that when an individual is fatigued or bored, activation of postsynaptic D1 and α2A receptors is insufficient due to the lack of DA or NE release. This mechanism leads to an individual being easily disturbed and impulsive. If too much of those NTs are released under stressful conditions, it can lead to overstimulation of receptors, resulting in inattention and responses. Focused, organized, and attentive actions and thoughts are produced after a modest stimulation of postsynaptic receptors of DA and NE. In ADHD individuals, tonic pool is thought to be reduced, which can result in more phasic release of DA, causing behavioral issues including inattention, hyperactivity and impulsivity. By antagonizing DAT-1 and NET, stimulants can boost the tonic pool and lessen the phasic release fired by action potential, which might be the underlying issue in ADHD. AP, action potential; DA, dopamine; NE, norepinephrine; PFC, prefrontal cortex, DAT-1, dopamine transporter-1; NET, norepinephrine transporter; Dn receptor, Dopamine-n receptor (n = number); PFC, prefrontal cortex.

The precise mechanisms underlying ADHD have not yet been clearly established. However, it has been shown that a reduced volume or functionality of the brain’s gray and white matter might lead to deficits of attention, cognition, processing response speed, motor planning, and other behavioral problems shown in ADHD [15,16]. Recent research studies have proposed that the cerebellum, caudate, and prefrontal cortex (PFC) as primary areas related to deficits in ADHD are interconnected together as a neuronal network for regulating attention, thoughts, behavior, actions, and emotions [17,18]. The network activity between these areas is mediated by neurotransmitters (NTs), norepinephrine (NE), and dopamine (DA) via multiple receptors in presynaptic or postsynaptic neurons [17,19,20,21,22]. Figure 1 demonstrates the integration of the hyper- and hypo-active catecholamine hypothesis of ADHD [23].

Several studies have measured the plasma levels of monoamines in an attempt to explicate the underlying biological mechanisms involved in ADHD [24]. However, the results are inconclusive, so further investigation is required. Another possible way to explain the underlying pathophysiology of ADHD is the alteration of the tryptophan metabolic pathway (TMP) [25,26]. Tryptophan (TRP) is the most prevalent amino acid with a significant role in the biosynthesis of proteins in humans and animals. The metabolic fate of TRP is bifurcated into two pathways: the kynurenine pathway (KP) and the serotonin pathway (SP) [27,28]. Several studies using animal/human models have shown the role of tryptophan (TRP) metabolism in ADHD by demonstrating levels of TRP metabolites such as kynurenine (KYN), kynurenic acid (KYNA), and 3-hydroxynurenin (3-HK) [29]. TRP and its metabolites play an important role in mitigating diverse disorders ranging from cancer to psychiatric or neurological diseases [30,31,32]. Recent studies have demonstrated that KP can affect diverse biological systems. Therefore, the potential of using metabolites in KP as promising therapeutic biomarkers has attracted great interest from biomedical researchers [33]. Furthermore, the mechanisms linking KP to ADHD provide an excellent opportunity to establish new treatment for neuropsychiatric disorders [33,34].

Currently, there has been no clinically reliable biomarker for the diagnosis of ADHD, although several plausible inflammatory biomarker candidates have been suggested based on recent advances in biochemical and molecular biology [12]. Determining stable and reliable biomarkers to separate definite ADHD from normal behaviors that are not etiologically ADHD is highly desired to make less misdiagnosis.

From this point of view, the aim of the present review article was to summarize the potential inflammatory biomarkers and the underlying mechanisms, including detailed description of the kynurenine pathway, inflammatory-cytokine-mediated regulation of the kynurenine metabolism, dysregulation of the kynurenine pathway in ADHD, and potential biomarkers in ADHD.

## 2. The Kynurenine Pathway

The KP is involved in catabolic TRP degradation. It acts on glutamate receptors in the central nerve system and potentially regulates the essential mechanisms of ADHD by generating a number of neuro-active compounds collectively called kynurenines that could interact with neurotransmitter (NT) receptors in the central nerve system (CNS) [35]. This pathway takes place in the liver, kidney, and brain of mammals such as human [36]. Although the liver and kidney show the highest concentrations of enzymes, all primary enzymes are also found in the brain [37,38]. Kynurenine metabolism happens all over brain cells despite various annexes of the pathway being segregated into specific cell types [39].

**Figure 2:**
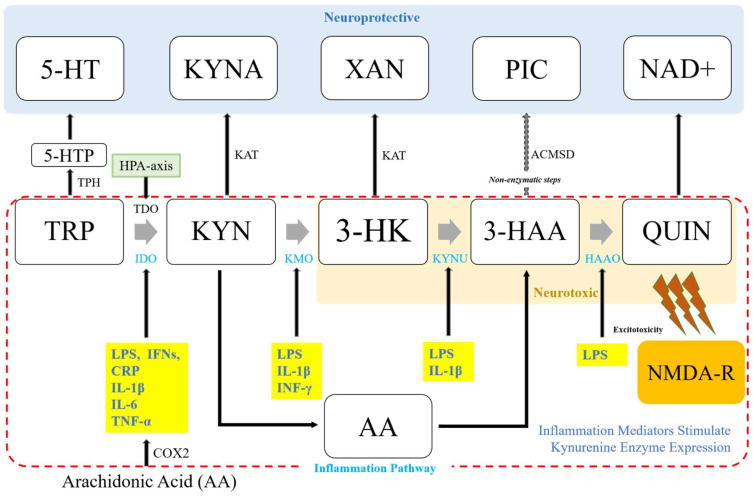
Schematic connection between the kynurenine pathway and nflammation. The kynurenine pathway (KP) bifurcates into two distinct branches modulated by the availability of l-kynurenine in the brain: KATs, and KMO. In addition, a variety of inflammation-related mediators known to affect enzyme expression can regulate the metabolism of kynurenine by adjusting substrate availability and metabolite formation, preferring the KMP direction of the pathway with immune-related pathological conditions. TRP, tryptophan or L-tryptophan; TDO, TRP 2,3-dioxygenase; 5-HT, serotonin; KYN, kynurenine or l-kynurenine; 5-HTP, 5-hydroxytryptophan; HPA-axis, hypothalamic-pituitary-adrenal axis; KYNA, kynurenine acid; 3-HK, 3-hydroxykynurenine; AA, anthranilic acid; XA, xanthurenic acid; NAD+, nicotinamide adenine dinucleotide; PIC, picolinic acid; 3-HAA, 3-hydroxyanthranilic acid; ACMSD, aminocarboxymuconate semialdehyde decarboxylase; QUIN, quinolinic acid; NMDA-R, N-methyl-D-aspartate receptor; IDO, indoleamine-2,3-dioxygenase; KAT, kynurenine aminotransferase; KMO, kynurenine 3-monooxygenase; KYNU, kynureninase; HAAO, 3-hydroxyanthranilic acid oxidase; LPS, lipopolysaccharide; BCG, bacillus Calmette-Guerin; IFNs, interferons; TNF, tumor necrosis factor; IL, interleukin; TNF-α; tumor necrosis factor alpha, IFN-γ, interferon gamma; COX2, cyclooxygenase 2.

The KP pathway is initiated either by tryptophan-2,3-dioxygenase (TDO) in the liver to open the TRP indole ring, or indole-2,3-dioxygenase (IDO) in the brain to produce an instable metabolite of *N*-formylkynurenine [40,41] (Figure 2). The conversion of N-formylkynyrenine to L-kynurenine (KYN) is then followed. KYN is a substrate of various enzymes, including kynureninase (KYNU) for catalyzing the production of anthranilic acid (AA), kynurenine aminotransferases (KATs I-IV) for kynurenine acid (or kynurenic acid, KYNA), and kynurenic 3-monooxygenase (KMO) for 3-hydroxykynrenine (3-HK). From L-KYN, the pathway bifurcates into two distinct branches often called “neuroprotective” and “neurotoxic” arms. The neuroprotective arm is modulated by KAT, whereas the neurotoxic part is modulated by KMO.

Brain kynurenine metabolism occurs mostly in glial cells. KMO, KYNU, and 3-hydroxyanthranillic acid oxidase (3-HAAO) can regulate the formation of L-KYN in microglia, resulting in the formation of AA, 3-HK, 3-HAA, and quinolinic acid (QUIN). QUIN is excitotoxic at NMDA glutamate receptors. It has a synergistic effect with 3-HK in generating oxidative stress [42]. In astrocytes, L-KYN can be metabolized by KATs alternately. KAT II is the predominant subtype in brains of humans and rats [43]. KATs can catalyze L-KYN to KYNA, a glutamate neurotransmission inhibitor and a possible antagonist at nicotinic α7 receptors. In conjunction with these roles, KYNA can interact with arylhydrocarbon receptors and GPR35 [19,44]. In such a way, kynurenine-derive neuro-active compounds have multiple receptor targets. Further research is needed to clearly demonstrate their endogenous function. A third possible degradation modulated by both KATs and KMO is xanthurenic acid (XA). Although not much is known about XA, it might play a role in modulating glutamatergic neurotransmission by activating Group II metabotropic glutamate receptors (mGlu2 and mGlu3) or inhibiting vesicular glutamate transporters, indicating that it could also modulate glutamate neurotransmission by impacting presynaptic release [45,46].

During the last decades, the underlying regulatory mechanism of kynurenine metabolism has been extensively investigated as it has a role in CNS disorders [47,48,49]. As ‘’neuroprotective” and “neurotoxic” branches of the pathway, KATs and KMO, respectively, can balance the production of KYNA:QUIN which is essential in psychiatric and neuropsychiatric diseases. Some kynurenine metabolites can pass through the blood brain barrier, indicating that CNS levels of kynurenines are mostly regulated by peripheral enzyme activities [50]. However, a large neutral amino acid transporter can actively convey kynurenine into the brain [51]. Most kynurenine which is metabolized into KYNA and QUIN is from the periphery under normal physiological conditions [52]. After systemic inflammation, nearly all kynurenine in the CNS is derived from the periphery, at which IDO expression is greatly increased [53]. However, the direct induction of CNS inflammation can result in over 98% of kynurenine from local synthesis in the brain [52].

To the best of our knowledge, only three research groups have investigated serum levels of kynurenines in patients with ADHD. Evangelisti et al. [10] have reported the most recent measurements [25,54], showing that serum concentrations of kynurenic acid (KA), xanthurenic acid, and anthranilic acid are lower, while tryptophan and kynurenine concentrations are significantly higher in children with ADHD than in healthy controls. They found that the existence of ADHD was significantly related to low AA levels and high TRP levels in a human logistic regression model [10]. The following section summarizes the interaction between inflammatory mediators and their underlying mechanisms regulating the KP.

## 3. Inflammatory Cytokine-Mediated Regulation of Kynurenine Metabolism

### 3.1. Indoleamine 2,3-Dioxygenase (IDO) and Inflammation Mediators

The first step of TRP catabolism takes place with IDO and TDO, which are generally known to be differently modulated. While IDO is induced by pro-inflammatory cytokines during immune response, TDO is induced by glucagon and corticosteroids [55]. There is some evidence showing that other enzymes in the neurotoxic branches of the KP can be also induced by pro-inflammatory cytokines. However, IDO regulation by interferon (IFN)-γ has been investigated most widely.

While IFN- γ is regarded as the primary IDO inducer, there is evidence showing that the expression of IDO can be induced independently of IFN- γ [56,57,58]. In vitro data using HTP-a cells, i.e., a human monocytic cell line, have shown that LPS-induced IPO activation is mediated by an IFN-γ-independent mechanism, including the synergistic effects of TNF-β, IL-6, and IL-β 1 [57]. Connor et al. [56] have also suggested that IFN-γ might not be necessary for LPS-induced IDO expression in mixed glia cultures harvested from neonatal rat cortex. In human progenitor cells of the hippocampus, IL-β treatment does not upregulate TDO, but greatly increases the expression of IDO transcript. This supports the fact that IL-β can elevate functional levels of IDO enzyme [58].

Experiments investigating the role of anti-inflammatory cytokines in IDO expression have shown limited and often conflicting results. This might be due to differences in the models used and experimental conditions applied. For instance, IL-10 as one of the major anti-inflammatory cytokines decreased LPS-mediated IDO protein expression in a dose-dependent manner. However, IFN-γ-mediated IDO protein expression was increased by IL-10 in mouse bone marrow-derived dendritic cells (BMDCs) [59]. This inconsistency may propose that anti-inflammatory cytokines such as IL-10 can differentially regulate the distinct mechanisms of IDO induction. However, it has not been demonstrated whether this occurs in the brain. Notably, IFN-γ-treated IDO expression in a transformed mouse neuronal cell line was suppressed by IL-10 [60]. In addition to the case of IL-10, studies on human monocytes and fibroblasts have suggested that IL-4 can inhibit IDO mRNA induction and IDO activity by IFN-γ. Opposed to this, a study using mouse microglia cells reported that IL-4 can enhance IFN-γ induced IDO mRNA expression, which is diminished by IL-4 antiserum addition [61]. Along with IL-4, IL-13 which utilizes the same receptor subunit in signaling can potentiate IFN-γ treated IDO mRNA expression in mouse microglia cultures [61]. Collectively, these findings indicate that responses to anti-inflammatory cytokines in microglia and peripheral myeloid cells are different.

Other than those anti-inflammatory cytokines, pro-inflammatory cytokines (such as TNF-α and IL-1β) and toll-like receptor (TLR) agonist (such as LPS) can synergistically potentiate IFN-γ-treated IDO expression [62]. Moreover, TNF-α can synergistically induce IDO expression with IFN-γ by increasing NF-κB-dependent IRF-1 expression and STAT- nym1 activation [62]. Synergistic IDO induction by TNF-α and IFN-γ occurs in primary mouse microglial cells. The mechanism has been utilized in a research model of inflammation-related depression [53].

IFN-γ-independent IDO induction is supported by studies using primary mouse microglial cells demonstrating that IFN-γ mRNA is not detectable whereas IDO mRNA levels are increased after LPS stimulation [19,56]. Other studies using THP-1 cells have indicated that LPS-boosted L-KYN production does not occur with IRF-1 or STAT-1 binding activation, but is lessened by p38 and NF-κB inhibitors [57]. To sum up, IDO induction stimulated by LPS in monocytes is IFN-γ-independent and related to NF-κB as well as stress-activated mitogen-activated protein kinases (MAPK) including JNK and p38 [19,56,57]. Although subsequent mechanisms between JNK or p38 and IDO induction in response to LPS stimulation have not been clearly established, AP-1 factors are conventional substrates of MAPKs. They have critical roles as inflammation-related gene transcription regulators [19].

### 3.2. Kynurenine-3-Monooxygenase (KMO) and Inflammation Mediators

Similar to IDO, pro-inflammatory stimuli may activate KMO enzymes downstream of the pathway. After the systemic inoculation of LPS, KMO expression is induced in rat brain [56]. KMO is also induced in both IFN-γ treated immortalized murine microglia (N11) and macrophage (MTs) cells. However, KYNU is induced only in MT2 whereas 3-HAAO is not affected [63]. In human progenitor cells of hippocampus, transcriptional levels of KMO and KYMU are upregulated following IL-1β [58].

### 3.3. Kynurenine Aminotransferases (KATs) and Inflammation Mediators

Compared to the expression levels of IDO and other kynurenine enzymes in the neurotoxic branch of the KP, KAT expression is neither elevated nor changed in response to pro-inflammatory stimuli. Systematic LPS inoculation of LPS causes no change in KAT II in rat brain cells [56]. In immortalized murine microglia (N11) and macrophage (MTs) cells, KAT shows constitutive expression. IFN-γ treatment shows no effect on KAT activity [63]. In human progenitor cells of hippocampus, IL-1β treatment downregulates only KAT I and III, showing no effect on KAT II [58].

## 4. Genetic Links between Inflammation and Kynurenine Metabolism in ADHD

Genetic studies have supported that gene polymorphisms are linked to the inflammatory pathway in ADHD. In a total of 398 subjects, Smith et al. [30] evaluated a set of 164 single-nucleotide polymorphisms (SNPs) from 31 candidate genes and found that two SNPs in the ciliary neurotrophic factor receptor (*CNTFR*) were associated with the severity of ADHD inattentive symptom. Odell et al. [64] conducted a population-based association study with 546 ADHD patients vs. 546 controls and proposed an association between *CNTFR* and ADHD in both children and adults. They also reported an association between ADHD and major histocompatibility complex genes, demonstrating the role of inflammation and autoimmunity in this disorder. However, recent findings of a genome-wide association meta-analysis have failed to replicate these results [65].

Another genome-wide association study for 478 ADHD patients and 880 controls has suggested no significant SNPs [66]. However, a pathway analysis has revealed an association of ADHD with SNPs involved in gene expression regulation, cell adhesion, and inflammation [30]. One study has inspected the genomic overlap between ADHD and other psychiatric disorders in 318 individuals, including 93 who were diagnosed with ADHD, and found a similar inflammation-related genetic signature between ADHD and depression [67]. Segman et al. [68] evaluated IL-1 receptor antagonist gene variable number tandem repeat polymorphism in a risk population of ADHD. As IL-1 is known to regulate murine catecholaminergic transmission, it was selected for the study. Segman et al. [68] found an association between a four-repeat allele and an increased risk for ADHD and an association between a two-repeat allele and a decreased risk. However, they failed to reproduce the same results with a larger sample later [69].

While there is a good number of genetic studies supporting the association between inflammation and ADHD, there are high variations for methodologies applied in each study. Highly heterogeneous genetic features and clinical manifestation might take part in the observed variation. Currently, there is no consensus about which inflammatory-related genes precede ADHD. Future research on populations with heterogeneous features might provide more conclusive findings.

## 5. Dysregulation of the Kynurenine Pathway in ADHD

A delay in the development of cortical maturation may cause evident deficits in neuropsychological performances in ADHD [16]. Although the etiology of this delay is unknown, impaired glial supply to support energy for neuronal activity has been suggested to have a contribution. A recent study on ADHD proposed that patients may carry subsyndromal immunological imbalances such as increased serum IFN-γ and IL-13 levels. It also demonstrated a decreased 3-HK despite normal levels of L-KYN [26]. Compared to medicated subjects, the alteration of pro-inflammatory cytokine production level and kynurenine metabolism showed a trend toward normalizing in medication naïve subjects. An impaired 3-HK production might be predisposed to reduced activation of microglia and hence impaired neuronal pruning that could bring in developmental delays. These reports might be congruous with early postulations about an imbalance of TRP metabolism in ADHD, suggesting that patients can produce excess serotonin, at least in peripheral compartments [70].

Although no report has directly explored cytokine and kynurenine profiles at the CNS level in ADHD, a few studies have tried to establish the association between these markers and behavioral endophenotypes by measuring their serum levels. Oades et al. demonstrated that levels of S100b are negatively associated with oppositional and conduct problems in ADHD [71]. Their study also demonstrated an inverse relationship between S100b and IL-10/IL-16 in children with ADHD. A subsequent study has reported that hyperactivity is strongly correlated with reduced S100b, while attention capacity may be related to IL-13 [26]. Increased kynurenine and IFN-γ (though reduced TNF-α) are related to faster reaction time, whereas TRP metabolism shows no relation with symptoms. Another study conducted by Oades et al. [54] demonstrated that increases in 3-HK and IFN-γ are linked to lower birth weight and shorter pregnancy in individuals with ADHD, which are associated with the severity of symptoms. This result was only partially congruent with former reports [26] of dysregulated cytokine production and kynurenine metabolism, where a decrease in 3-HF was found. Although these findings of peripheral cytokine and kynurenine system alterations are impressive, further research is required to elucidate whether these peripheral measurements might be interpreted as changes in the CNS compartment. In addition, a detailed analysis of cytokine levels and their relationship to the KP in the brain throughout the disease might be beneficial to research on developmental delay reported in ADHD patients.

## 6. Potential Inflammatory Biomarkers in ADHD

Recently, psychiatry research studies have measured and examined how individual cytokines known to be related to inflammatory processes are related to particular diagnostic categories and related phenotypes. Individual relationships of these markers with various mental disorders in perinatal and offspring outcomes, chronic states, and pre/post-treatment have been examined based on cytokines, C-reactive protein, hormones, neurotrophins, and so on. Some commonly investigated cytokine measures are summarized in Table 1 [12,40,72,73,74,75,76,77,78,79,80,81,82,83,84,85,86,87,88]. When interpreting reports of individual studies, it is crucial to consider the extent to which other factors affecting peripheral cytokines are accounted for in specific analyses. Compounding factors including age, sex, weight, smoking, childhood trauma, the timing of blood sampling, medical comorbidities, concurrent medication use, and severity of illness are example variables that should be but are not always indicated in studies. They are possible sources of discrepancy in results [82,89].

It has been reported that neuroinflammation might underlie the neurodevelopment of the immune system, resulting in changes in normal microglia, astrocytes, chemokines, cytokines, oxidative stress, and related metabolism in the first months or early years of life [92]. In children with maternal inflammatory and immune system disruption, an increased profile of ADHD risk has been observed [93]. Gustafsson et al. [94] suggested that maternal serum levels of IL-6, TNF-α, and monocyte chemoattractant protein-1 (MCP-1) are possible markers of ADHD risk, which is the first human study providing evidence of an association between inflammation and brain developmental/behavioral defects.

Consistently, mechanistic evidence has been demonstrated by animal studies, which show maternal immune activation in offspring with ADHD [29]. During perinatal development, neurodevelopmental high inflammatory responses triggered by several mechanisms of environmental factors such as heavy metal exposures may increase ADHD risk conditionally [54].

Peripheral pro-inflammatory cytokines can cross the brain through humoral and neural pathways and maintain inflammatory responses via neuroimmune systems. Inflammation-related cytokine changes in the brain are known to cause neurotransmission changes in TRP metabolism and dopaminergic pathways in the brain, similar to those seen in patients with ADHD [71]. At this point, prenatal exposure to inflammation may restrain brain development resulting from structural changes in the volume of gray matter that can cause permanent neural circuits to fail to mature or bring neuroendocrine changes, thus elevating the risk of ADHD [95]. In addition, the interaction of the HPA axis with a chronic increase in cytokine signals in the immune system during developmental processes is associated with the pathogenesis of ADHD [96]. However, these serological changes in inflammatory cytokines tend to wane or become reversed along with advances in the age of ADHD patients. Therefore, the age of subjects in studies measuring potential inflammatory biomarkers of ADHD should be included when interpreting study results.

In recent studies, various inflammatory cytokines in the central and peripheral samples have been proposed as feasible potential biomarkers of ADHD risk. Table 2 lists potential inflammatory biomarkers of ADHD risks suggested for youth and adult ADHD patients.

Chang et al. [75,76] evaluated CRP levels and found that its levels are elevated in youth with ADHD compared to those in healthy youth. Concurrently, TNF-α levels have been reported to be lower in children with ADHDsince IL-6 is known to have a suppressive effect on TNF-α production [104]. They mutually counter-regulate each other during early immune responses. Yang et al. [87] evaluated serum CRP and other cytokines in individuals aged 5 to 55 years and provided sub-analysis results, showing that reported CRP levels were elevated in the adult group. They analyzed scores of Difficulties in Emotion Regulation Scale (DERS-16) and CRP levels and found that higher CRP levels were associated with lower DERS-16 scores.

Oades et al. [71] investigated serum levels IL-1β, IL-6, IL-10, IL-13, IL-16, and TNF-α in 21 children (mean age: 8.9 ± 1.4 years) with ADHD who are treatment-naïve compared to the same number of controls (mean age: 12.6 ± 2.1 years). Lower IL-13 levels were seen in the ADHD medication-naïve group compared to those in the control. With the same sample, the authors further analyzed the correlation between levels of cytokines and symptom scores. An increase in IL-13 was associated with increased inattention symptoms. A high IL-16 level was associated with an increase in hyperactivity impulsive symptoms and positively related to motor activity. Another study [54] reported that an increased IL-16 level in the ADHD group was related to poor infant health. Numerous reports have shown that increased level IL-6 levels in ADHD are associated with an increased risk of ADHD in children [71,75,97]. However, this evidence is diluted in advanced age groups of ADHD patients [99,100].

Misiak et al. [86] conducted a systematic review on possible peripheral blood inflammatory markers in ADHD patients and found no association with IL-6 or IL-10 in ADHD. However, individual case-control studies conducted by Donfrancisco et al. [98] have found that IL-6 and IL-10 levels are elevated in children with ADHD. The heterogeneity of subjective research in the systematic review might be one reason for the diluted result. A systematic review conducted by Misiak et al. [86] has also found that lower TNF-α is meaningful in both adult and youth ADHD patients.

Chang et al. investigated inflammatory biomarkers in ADHD and revealed that morning salivary cortisol levels were lower in youths with ADHD than in healthy controls [75,76]. This result was reproduced in the study of Llorens et al. [101] recently, which also reported lower morning cortisol levels in youths with ADHD with consistently high levels of hyperactivity and inattention symptoms since childhood. A previous research by Isaksson et al. [102] found a similar result between childhood trauma and ADHD symptoms regarding morning cortisol levels, with children with ADHD showing a positive correlation between childhood adversity and cortisol increase after awakening. Scassellati et al. [103] performed a systematic review on potential biomarkers in adults with ADHD and reported lower salivary cortisol levels from a meta-analysis.

Although the most recent research on ADHD biomarkers has suggested the possibility of finding more objective forms of diagnostics compared to the current diagnostic criteria in clinical use today, it remains unclear how these discrete markers are associated with diverse clinical manifestations and different populations that persistently confound research on ADHD. A few observational studies have evaluated different variables without adjusting or controlling confounders to investigate the role of inflammation in the pathophysiology of ADHD, yielding conflicting results. Further research overcoming these limitations of previous research studies performed so far is needed.

## 7. Summary

The goal of this review was to explore the possible underlying etiopathophysiology of KP in the regulation of ADHD. This reviewed summarized the current knowledge on the range of possible peripheral inflammatory biomarkers. Uncertainty remains as to whether neuroinflammation is a cause or consequence of the risk of developing ADHD. However, some researchers have demonstrated that the KP and subsequent neuroinflammation have a certain degree of association with the state of ADHD. Searching for reliable peripheral inflammatory biomarkers is of great interest in terms of eliminating uncertainty and overcoming diagnostic and treatment difficulties in the clinic. Although a solid neuroimmunological basis has been established through recent neuroimmunological advances, there is a need for further studies to determine how changes in inflammatory markers are related to disease status or whether these markers can be used to detect the development of ADHD and predict its progression and response to treatment.

## Figures and Tables

**Table 1:** Commonly investigated inflammation-related cytokines in neuropsychiatric disorders.

Category	Protein Designation	Name	Major Function
General	CRP	C-reactive protein	Acute-phase protein produced in response to acute and chronic inflammation Produced as a result of increasing pro-inflammatory cytokines (IL-1 and IL-6) and lipopolysaccharides
Pro-inflammatory cytokine	IFN-γ	Interferon gamma	Secreted by lymphocytes and is a potent activator of macrophages Critical to both innate and adaptive immunity
	IL-1β	Interleukin 1 beta	Induces prostaglandin synthesis, neutrophil and T-cell activation, cytokine production, B-cell activation and antibody production Utilized as a biological response modifier in cancer therapy
	IL-6	Interleukin 6	Pyrogenic, acute-phase response mediator, stimulating acute-phase protein synthesis and production of neutrophils Supports the growth of B-cell
	IL-8	Interleukin 8	Chemotactic factor in the recruitment of neutrophils and other immune cells to the site of inflammation
	IL-10	Interleukin 10	A potent anti-inflammatory cytokine that plays a central role in limiting pro-inflammatory cytokines and maintaining tissue homeostasis
	IL-18	Interleukin 18	A pleiotropic cytokine produced mainly by antigen-presenting cells, plays a key role in autoimmune, inflammation, and infection Enhances cytotoxic activity and the proliferation of CD8+ T and NK cells Stimulates the production of other cytokines including IL-13
	TNF-α	Tumor necrosis factor alpha	Produced by macrophages during acute inflammation and plays a role for cell necrosis or apoptosis
	TNF-β	Tumor necrosis factor beta	Involved in autoimmune disorders, mediating the inflammatory demyelination process
Adapted from Zlotnik (2012) [90] and Bishop et al. (2022) [91].			

**Table 2:** Potential inflammatory biomarkers in youth and adult ADHD patients.

Biomarkers	Youth	References	Adult ^1^	References
CRP	↑	[75,76]	↑	[87]
IL-1β	↓	[71]	↔	[87]
IL-6	↑	[71,97,98]	↔	[86,99,100]
IL-10	↑	[71,75,98]	↔	[86]
IL-13	↑	[71,75]	↔	[86]
IL-16	↑	[54,71]		
TNF-α	↓	[71,86]	↓	[86]
Cortisol ^2^	↓	[75,76,101,102]	↓	[103]

^1^ Compared to controls. ^2^ Cortisol: Morning salivary cortisol.

## Data Availability

Not applicable.
